# Establishment of reverse genetics systems for Colorado tick fever virus

**DOI:** 10.1371/journal.ppat.1012921

**Published:** 2025-02-14

**Authors:** Shohei Minami, Ryotaro Nouda, Katsuhisa Hirai, Zelin Chen, Tomohiro Kotaki, Yuta Kanai, Takeshi Kobayashi

**Affiliations:** 1 Department of Virology, Research Institute for Microbial Diseases, Osaka University, Suita, Osaka, Japan; 2 Center for Infectious Disease Education and Research, Osaka University, Suita, Osaka, Japan; 3 Center for Advanced Modalities and DDS, Osaka University, Suita, Osaka, Japan; SUNY Upstate Medical University, UNITED STATES OF AMERICA

## Abstract

The Colorado tick fever virus (CTFV), which has 12-segmented double-stranded RNA genomes, is a pathogenic arbovirus that causes severe diseases in humans. However, little progress has been made in the analysis of replication mechanisms and pathogenicity. This virological constraint is due to the absence of a reverse genetics system for CTFV; therefore, we aimed to establish the system. Initially, the efficacy of CTFV replication was investigated in various cell lines. CTFV was found to grow in many cell types derived from different hosts and organs. Subsequently, BHK-T7 cells stably expressing T7 RNA polymerase were transfected with plasmids encoding each of the 12 CTFV gene segments, expression plasmids encoding all CTFV proteins, and a vaccinia virus RNA-capping enzyme. Following transfection, the cells were co-cultured with Vero or HeLa cells. Using this system, we rescued monoreassortants and recombinant viruses harboring peptide-tagged viral proteins. Furthermore, an improved system using Expi293F cells expressing T7 RNA polymerase was established, which enabled the generation of recombinant reporter CTFVs. In conclusion, these reverse genetics systems for CTFV will greatly contribute to the understanding of viral replication mechanisms, pathogenesis, and transmission, ultimately facilitating the development of rational treatments and candidate vaccines.

## Author summary

CTFV is an important zoonotic arthropod pathogen that causes CTF in humans through tick-bite transmission. The absence of a reverse genetics system for CTFV has limited advancements in research. To overcome this limitation, we aimed to establish a reverse genetics system for CTFV. We first examined the replication capacity of CTFV in various cell lines. Using this information, a reverse genetics system was established by transfecting BHK-T7 cells with 12 rescue plasmids and numerous enhancers, followed by co-culture with CTFV-sensitive cells. Further improvement of the CTFV rescue system was conducted using Expi293F cells expressing T7 RNA polymerase, which was highly sensitive to CTFV infection. These systems were used to generate several recombinant viruses for CTFV research. The systems established in this study provide a valuable platform for studying the biology of segmented viruses and developing strategies to prevent and control CTFV infections.

## Introduction

The *Coltivirus* genus, belonging to the *Reoviridae* family, is characterized by its 12-segmented double-stranded RNA (dsRNA) genomes [1]. The genus includes several virus species, including the Colorado tick fever virus (CTFV). CTFV causes Colorado tick fever (CTF) in humans and was first isolated in North America between the late 1920s and early 1930s [2–5]. Subsequently, the Eyach virus was isolated from ticks in northwestern France in 1976 [6,7]. Furthermore, antibodies against this virus were detected in a patient with meningoencephalitis, suggesting its potential pathogenicity in humans [6]. Recently, the Kundal virus was isolated from ticks in India [8], and the Tai Forest reovirus was isolated from a bat in Côte d’Ivoire [9]. Moreover, the Tarumizu tick virus (TarTV) has been isolated from ticks and a raccoon dog in Japan [10–12]. Other unclassified coltiviruses have also been reported [13–17]. Although viruses of the genus *Coltivirus* have been found in various regions, their replication mechanisms and pathogenicity remain unclear.

The distribution of CTFV is primarily confined to the surrounding region of the Rocky Mountains of the United States and Canada, owing to the limited habitat of its main carrier tick species, *Dermacentor andersoni* [7]. The major symptoms of CTF are high fever, chills, headaches, body aches, and fatigue, closely resembling influenza virus infections [7,18,19]. In rare cases, patients may present with severe central nervous system disorders [20]. CTFV infections continue to occur in these regions [7,19–21]. The oldest CTFV strain isolated from a patient with CTF was designated as the Florio strain and is widely used in CTFV studies as a prototype strain [2–5]. Although CTFVs have been isolated from humans, rodents, and ticks, with ticks being the main natural hosts and transmission from ticks to mammals occurring via tick bites [20], there have been no reports of transmission among other mammalian species. The role of mammalian species in transmission remains unclear because viral transmission via tick bites depends on the viral load in animal sera. Moreover, the susceptibility of various animals to CTFV has not been determined. Therefore, some species could be dead-end hosts, whereas others are natural hosts.

Each genomic segment of CTFV encodes a viral structural or nonstructural protein. Although the functions of several of these proteins are unknown, functional analyses have been conducted on some of them. VP7, encoded by segment 7, is a highly antigenic protein, and it is used as an antigen for the enzyme-linked immunosorbent assay [22]. Segment 9 encodes VP9 and VP9’, which are regulated by a stop codon readthrough sequence [23]. Apoptosis is induced in cells infected with CTFV [24]. Furthermore, CTFV can infect hematopoietic cells, especially erythrocytes, suggesting that the virus can be transmitted through tick blood-sucking [25–27]. However, the mechanisms underlying CTFV infection and pathogenicity remain unknown owing to a lack of reverse genetics systems for this virus.

Reverse genetics systems, allowing for the manipulation of viral genomes, have been developed for many viral species and have greatly contributed to our understanding of viral biology. Reverse genetics systems for the *Reoviridae* family, containing 9–12 multi-segmented dsRNA genomes, have been recently established. In 2007, the first entirely plasmid-based reverse genetics system was developed for mammalian orthoreoviruses (MRVs) with 10-segmented dsRNA genomes [28]. Subsequently, DNA- or RNA-based reverse genetics systems were developed for dsRNA viruses with 10-segmented genomes, including those for bluetongue virus, African horse sickness virus, epizootic hemorrhagic disease virus, Nelson Bay orthoreovirus, and novel duck reovirus [29–36]. In 2017, an entirely plasmid-based reverse genetics system was developed for rotaviruses with 11-segmented dsRNA genomes [37]. The development of a reverse genetics system for dsRNA viruses with 12 segments in their genomes was delayed but was finally developed for TarTV, a member of the *Coltivirus* genus, in 2021 [12]. The TarTV rescue system is expected to be a useful tool for understanding coltivirus biology. However, the pathogenicity of TarTV in mammals remains unknown, and a reverse genetics system for pathogenic viruses with 12-segmented genomes, such as CTFV, is required to understand the pathogenicity of coltiviruses.

In the present study, we aim to develop reverse genetics systems for CTFV, which will serve as powerful tools for advancing basic and applied research on pathogenic 12-segmented dsRNA viruses.

## Results

### Growth of CTFV strains

**Fig 1:**
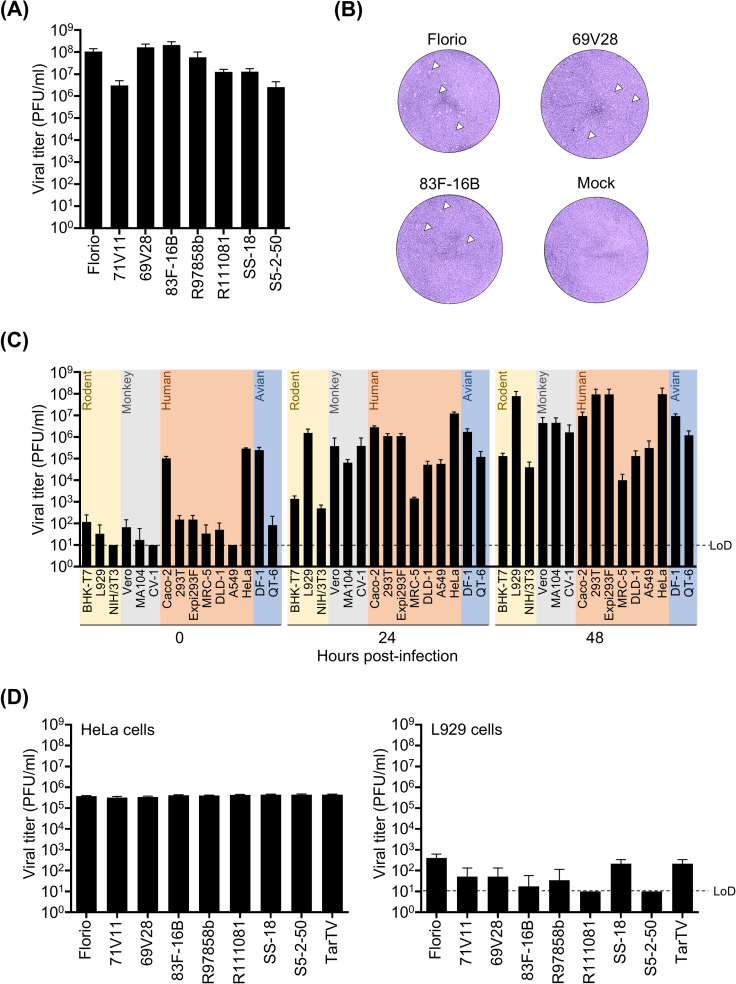
Growth of Colorado tick fever virus (CTFV) strains in various cell lines. **(A)** Vero cells were infected with eight CTFV strains at a multiplicity of infection (MOI) of 0.01. The samples were collected 72 hours post-infection (hpi). **(B)** Plaque formations by the CTFV strains in Vero cells are shown. The cells were fixed and stained 7 days post-infection. The high-resolution picture was taken using a Cytation 5 Cell Imaging Multimode Reader and stitched with each picture is shown as a figure. The plaques are indicated with white arrow heads. **(C)** Various cell lines derived from rodents (yellow), monkeys (gray), humans (orange), and avian (blue) infected with the CTFV Florio strain at an MOI of 0.01. The samples were collected at 0, 24, and 48 hpi. **(D)** HeLa and L929 cells were infected with the CTFV Florio strain at an MOI of 0.01. The samples were collected at 0 hpi. The 0 hpi sample was defined as collection after infection for 1 h, followed by washing the cells and soaking them in complete medium. The viral titers of frozen–thawed samples were measured by performing a plaque assay. The limit of detection (LoD) is indicated as a broken line. The experiments were repeated at least twice to confirm the result. Three biological and two technical replicates were conducted for each independent repeat.

We initially investigated the growth of eight CTFV strains (S1 Table) in monkey kidney Vero cells, which were previously used to establish a reverse genetics system for TarTV via co-culture with transfected cells [12]. This approach aimed to identify a highly proliferative strain suitable for the development of the reverse genetic system. The Florio, 69V28, and 83F-16B strains, isolated from CTF patients [2,38], reached titers of 1 × 10^8^ plaque forming unit (PFU)/ml in Vero cells at 72 hours post-infection (hpi) (Fig 1A). Florio was the first strain isolated, is a well-characterized prototypic strain [2–5], and showed high titer in Vero cells (Fig 1A). It formed clearer plaques (Fig 1B), and thus, we selected this strain to establish a reverse genetics system. To further understand the virologic characteristics of the CTFV Florio strain, we investigated its growth in various cell lines, including rodent, monkey, human, and avian cell lines, in addition to Vero cells. Although the Florio strain grew in baby hamster kidney BHK-T7 cells stably expressing T7 RNA polymerase, which are often used to establish a reverse genetics system for various RNA viruses, including dsRNA viruses, its viral titer in BHK-T7 cells at 48 hpi was lower than that in Vero cells (Fig 1C). Moreover, mouse fibroblast L929 cells, human colorectal epithelial-like Caco-2 cells, human kidney 293T cells, Expi293F cells, human cervical epithelial HeLa cells, and chicken fibroblast DF-1 cells facilitated the growth of the Florio strain better than Vero cells (Fig 1C). Notably, the infectious Florio strain was detected in Caco-2, HeLa, and DF-1 cells at 1 h after viral absorption (0 hpi), suggesting that CTFV could effectively bind these cell lines. To determine whether only the Florio strain could result in effective binding, we infected HeLa and L929 with seven other CTFV strains and a different *Coltivirus* species, TarTV, for 1 h at 37°C. Viral titers observed at 1 hour after virus absorption were compared between the two cell lines (Fig 1C). All CTFV strains and TarTV effectively bound HeLa cells, but not L929 cells (Fig 1D). These results indicate that several cell lines, including HeLa cells, are highly susceptible to CTFV infection during the early stages of infection and can be useful tools for establishing an efficient reverse genetics system.

### Establishment of a reverse genetics system for CTFV

**Fig 2:**
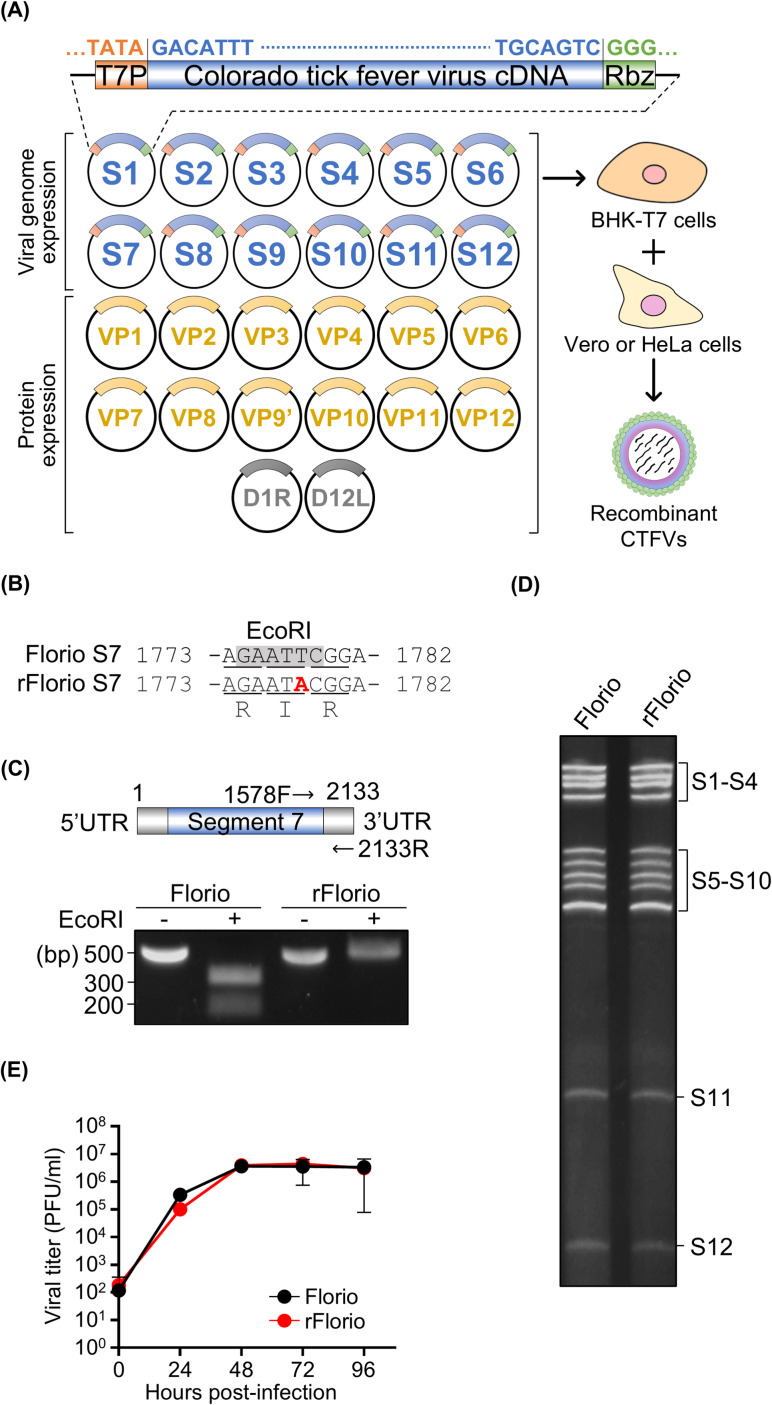
Generation and characterization of the recombinant Colorado tick fever virus (CTFV). **(A)** Schematic diagram of the reverse genetics system for CTFV established in this study. Each viral genome was cloned downstream of T7 RNA polymerase (T7P) and linked with the hepatitis D virus ribozyme (Rbz). **(B)** Nucleotide alignments of CTFV segment 7 (S7). The EcoRI digestion site is indicated by gray hatching. The synonymous mutation is indicated as a red, bold letter. **(C)** Schematic diagram of the amplicon obtained via RT-PCR, and images of the digested amplicons of S7 after electrophoresis. The black arrows indicate the primers. The electrophoresis was conducted using a 1% agarose gel. **(D)** Image of dsRNA genomes of CTFV strains after electrophoresis. The electrophoresis was conducted using a 6% polyacrylamide gel without SDS. **(E)** Growth kinetics of CTFV strains. Vero cells were infected with CTFV strains at a multiplicity of infection of 0.01, and samples were collected at each time point. The viral titers of frozen–thawed samples were measured by performing a plaque assay. The viral growth of rFlorio was compared with that of a parental CTFV Florio strain. The experiments were repeated at least twice to confirm the result. Three biological and two technical replicates were conducted for each independent repeat.

Based on existing reverse genetics systems for multi-segmented dsRNA viruses [12,29–36,39], each cDNA encoding the 12-segmented Florio strain genome was cloned into a plasmid containing a T7 promoter and hepatitis D virus ribozyme sequences (Fig 2A). In reverse genetics systems for multi-segmented dsRNA viruses, several viral proteins are overexpressed together with the introduction of viral genome transcription plasmids as rescue plasmids to enhance the rescue efficiency in BHK-T7 cells [12,30,37,39]. TarTV VP1, VP2, VP3, and VP4 proteins and vaccinia virus capping enzymes were overexpressed as rescue enhancers, along with 12 rescue plasmids in the TarTV reverse genetics system [12]. Consequently, we initially attempted to rescue recombinant CTFV based on the TarTV rescue system. However, recombinant CTFV could not be rescued in BHK-T7 cells transfected with the 12 rescue plasmids and expression plasmids encoding CTFV VP1, VP2, VP3, VP4, and vaccinia virus-capping enzymes. The system was further modified as follows: All CTFV protein expression vectors comprising each segment of the 12-segmented genome were co-transfected with 12 CTFV rescue plasmids and expression plasmids encoding vaccinia virus-capping enzymes into BHK-T7 cells (Fig 2A). At 6 hours post-transfection, the transfected cells were co-cultured with Vero cells. Following incubation for 21–28 days, lysates of co-cultured Vero cells were inoculated into fresh Vero cells. Subsequent incubation revealed cytopathic effects in the Vero cells inoculated with the lysates, suggesting that the recombinant viruses were successfully rescued from cells transfected with cloned CTFV cDNAs. To eliminate the possibility that the rescued recombinant CTFV Florio strain (rFlorio) was contaminated by native CTFV, we first confirmed the presence of a genetic marker in genome segment 7 (S7) of rFlorio. An artificial nucleotide mutation was introduced as a synonymous mutation in rFlorio S7 to modify the EcoRI restriction sequence (Fig 2B). The S7 genes of native Florio and rFlorio were then amplified via RT-PCR, and the amplified S7 fragments were treated with EcoRI. The amplified native Florio S7 fragment was digested by EcoRI, whereas the rFlorio S7 fragment was not (Fig 2C). These results indicated that rFlorio originated from the transfection of cloned viral cDNAs into BHK-T7 cells, confirming the absence of contamination by native CTFV. Next, we performed a comparative analysis between native Florio and rFlorio. The electrophoretic dsRNA genome pattern of rFlorio was identical to that of the native Florio strain (Fig 2D). There was also no significant difference in viral growth kinetics between wild-type Florio and rFlorio (Fig 2E). These results demonstrated that the recombinant virus had the same characteristics as the wild-type virus in Vero cells. Although recombinant CTFV could be rescued from cloned viral cDNAs, the rescue systems using Vero cells for co-culture with transfected BHK-T7 cells required a lengthy period (21–28 days) to successfully rescue rFlorio (Table 1). To generate recombinant CTFV more rapidly, we tested different cell lines for co-culture with the transfected cells in the CTFV reverse genetics system and investigated their effect on rescue efficiency. When HeLa cells were used for co-culture in the rescue system, the recombinant virus recovery time was shorter than that with Vero cells (Table 1). In contrast, the recombinant viruses could not be rescued when L929 cells were used (Table 1). Given that HeLa cells exhibited a higher infection efficiency than L929 and Vero cells in the early stages of CTFV infection (Fig 1C), the use of such cell lines with a high infection efficiency in the early stages of infection can considerably improve the rescue efficiency.

**Table 1:** Reverse genetics system for CTFV using co-culture with various cell lines.

Cell lines	rFlorio	Cultivation period (days)
Vero cells	Rescued	21–28
HeLa cells	Rescued	10–14
L929 cells	No rescue	> 30

### Characterization of CTFV monoreassortants and CTFV VP6

**Fig 3:**
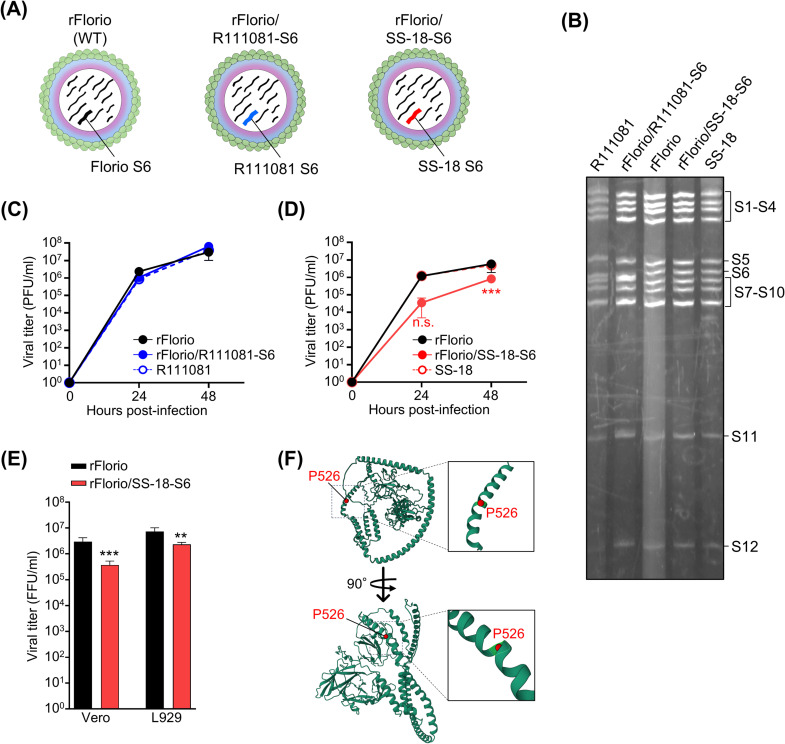
Characterization of recombinant Colorado tick fever virus (CTFV) reassortment between the Florio strain and other CTFV strains. **(A)** Schematic diagram of recombinant CTFV monoreassortants. The monoreassortants, in which segment 6 was reassorted between the Florio and R111081 strain (rFlorio/R111081-S6) or SS-18 (rFlorio/SS-18-S6), were generated using the reverse genetics system. **(B)** Image of dsRNA genomes of CTFV monoreassortants after electrophoresis. The electrophoresis was conducted using a 6% polyacrylamide gel without SDS. **(C, D)** Growth kinetics of CTFV monoreassortants. Vero cells were infected with CTFV strains at a multiplicity of infection (MOI) of 0.01. The samples were then collected at each time point. The viral titers of frozen–thawed samples were measured by performing a plaque assay. The viral growth of CTFV monoreassorted with R111081 **(C)** or SS-18 **(D)** strains was compared with that of a recombinant wild-type CTFV (rFlorio). **(E)** Growth of the CTFV monoreassortant in different cell lines. Vero and L929 cells were infected with CTFV strains at an MOI of 0.01, and samples were collected 48 hours post-infection. The viral titers of frozen–thawed samples were measured by performing a focus assay using an anti-VP5 antibody. **(F)** Predicted protein structural model of CTFV Florio strain VP6. The structure was developed using ColabFold v1.5.5. The P526 amino acid is highlighted by the red circle. Significant differences were determined by performing a two-way or one-way ANOVA. The experiments were repeated at least twice to confirm the result. Three biological and two technical replicates were conducted for each independent repeat. **0.005<*p*<0.01, ***p<0.005.

To determine whether the established system could be employed for a comparative analysis of different CTFV strains, we attempted to generate CTFV monoreassortants. We selected two different strains, R111081 and SS-18, isolated from humans and rodents, respectively, to generate monoreassortant viruses within the genetic backbone of Florio. Whereas the SS-18 strain is an ancestral isolate, the isolation of the R111081 strain was more recent than that of the Florio strain [40]. We used these strains to generate S6 monoreassortant viruses. The putative function of VP6, encoded by S6, is as an NTPase, which is important for viral replication [7]. S6-gene rescue plasmids derived from the R111081 and SS-18 strains were transfected with the other 11 Florio-based rescue plasmids and rescue enhancer plasmids into BHK-T7 cells. After co-culture and incubation with HeLa cells, monoreassortant viruses harboring the S6 gene of the R111081 (rFlorio/R111081-S6) and SS-18 (rFlorio/SS-18-S6) strains were successfully rescued (Fig 3A). Electrophoretic analysis of the viral dsRNA genomes revealed that the S6 gene of rFlorio/R111081-S6 migrated faster than that of the Florio strain (Fig 3B). Considering the S6 gene of rFlorio/SS-18-S6 had the same electrophenotype as that of the Florio strain, incorporation of the SS-18 strain S6 gene into segmented rFlorio genomes was confirmed via RT-PCR and Sanger sequencing of the viral dsRNA genome. rFlorio/R111081-S6 growth was similar to that of wild-type rFlorio (Fig 3C). Notably, the growth of rFlorio/SS-18-S6 was significantly decreased in Vero cells (Fig 3D). Considering the Florio and SS-18 strains were isolated from different hosts, humans and rodents, respectively, it is possible that VP6 is involved in the strain-specific differences in viral growth. To test this, viral growth in L929 cells was assessed. The growth of rFlorio/SS-18-S6 was decreased in L929 cells as well (Fig 3E), indicating that the reduced viral growth of rFlorio/SS-18-S6 is a strain-specific difference. The amino acid identities of VP6 between Florio and SS-18 and between Florio and R111081 strains were 99.9% and 90.1%, respectively. The amino acid sequences of the Florio and SS-18 strains differed only at position 526 (Florio: P526, SS-18: L526). In the structural prediction conducted using AlphaFold2 [41,42], 526 amino acid residues were determined to be located on the surface of CTFV VP6, comprising alpha helices (Fig 3F), suggesting that the 526^th^ amino acid residue of VP6 might be involved in the interaction with viral or host proteins.

### Generation of recombinant CTFV harboring peptide-tagged VP12

**Fig 4:**
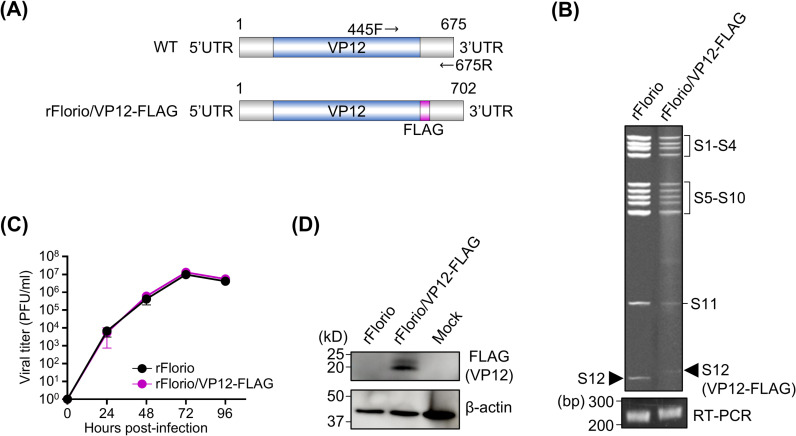
Characterization of the recombinant Colorado tick fever virus (CTFV) harboring peptide tag-fused VP12. **(A)** Schematic diagram of CTFV segment 12 encoding VP12. A recombinant CTFV harboring FLAG tag-fused VP12 (rFlorio/VP12-FLAG) was obtained by inserting a FLAG tag at the C-terminus of VP12. rFlorio/VP12-FLAG was generated using the reverse genetics system. The genome was amplified via RT-PCR using specific primers (black arrow). **(B)** Image of dsRNA genomes of rFlorio/VP12-FLAG after electrophoresis. The electrophoresis was conducted using a 6% polyacrylamide gel without SDS. Images of the amplicons of segment 12 after electrophoresis are shown below. **(C)** Growth kinetics of CTFV strains. Vero cells were infected with CTFV strains at a multiplicity of infection of 0.01. The samples were collected at each time point, and viral titers of frozen–thawed samples were measured by performing a plaque assay. The viral growth of rFlorio/VP12-FLAG was compared with that of a recombinant wild-type CTFV (rFlorio). **(D)** Western blotting of rFlorio/VP12-FLAG-infected cells. The FLAG tag-fused VP12 was detected using an anti-FLAG antibody. β-actin was detected as the loading control. The experiments were repeated at least twice to confirm the result. Three biological and two technical replicates were conducted for each independent repeat.

Using the developed CTFV reverse genetics system, we investigated the possibility of inserting foreign genes into the CTFV genome. To generate the desired recombinant virus, a FLAG-tag sequence was fused to the C-terminus of the VP12 protein encoded by the S12 gene (Fig 4A), and a recombinant CTFV strain harboring peptide-tagged VP12 (rFlorio/VP12-FLAG) was successfully generated. The electrophoretic pattern of the rFlorio/VP12-FLAG dsRNA genomes was identical to that of the wild-type Florio strain, except for the S12 gene (Fig 4B). The insertion of the FLAG-tag into the VP12 gene was also confirmed via RT-PCR and Sanger sequencing. The viral growth of rFlorio/VP12-FLAG was similar to that of rFlorio (Fig 4C). Vero cells infected with rFlorio/VP12-FLAG were analyzed via western blotting to confirm the expression of FLAG-tagged VP12. Clear bands corresponding to FLAG-tagged VP12 were detected at approximately 20 kDa, the correct size for native VP12 (Fig 4D). The localization of VP12 was analyzed using the recombinant CTFV harboring the FLAG-tagged VP12 by indirect fluorescent assay. The signal of FLAG-tagged VP12 had critically low brightness, resulting in unclear localization of VP12. These results indicate that CTFV can express foreign peptide sequences and that recombinant viruses expressing FLAG-tagged VP12 are useful for functional analysis of the VP12 protein.

### Generation of recombinant reporter CTFVs

**Fig 5:**
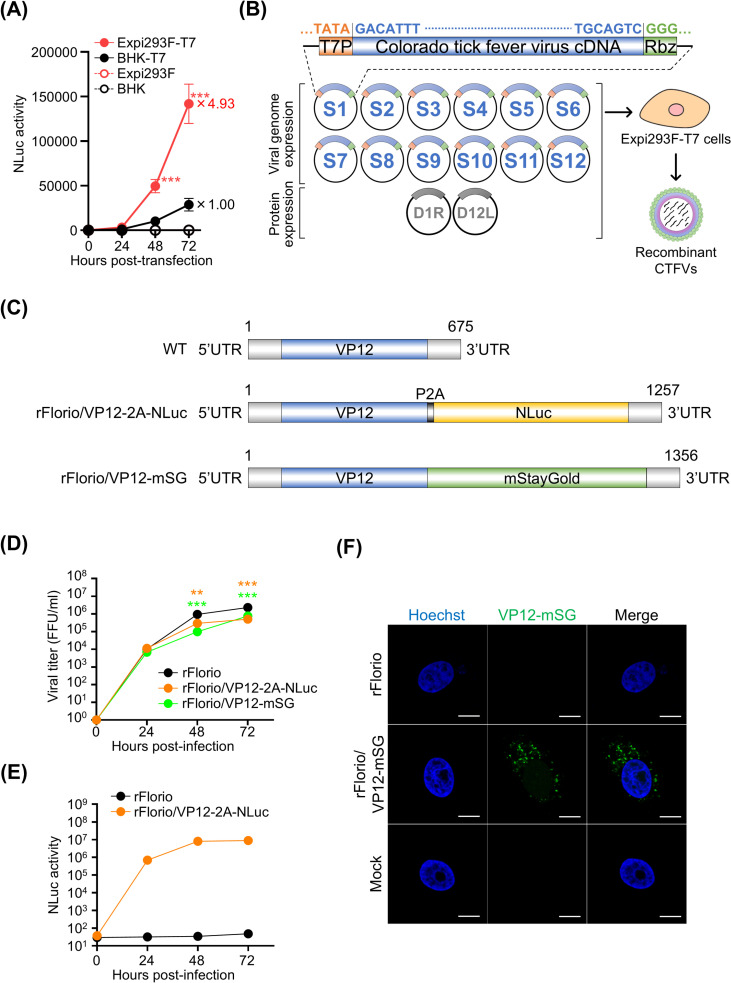
Optimization of the reverse genetics system and characterization of the recombinant reporter Colorado tick fever viruses (CTFVs). **(A)** Activity of T7 RNA polymerase in Expi293F-T7 cells. The cells were transfected with pT7-sNLuc plasmids encoding secreted NLuc gene. After incubation, the NLuc activity of the supernatant was measured. **(B)** Schematic diagram of the improved reverse genetics system for CTFV. Each viral genome was cloned downstream of T7 RNA polymerase (T7P) and linked with the hepatitis D virus ribozyme (Rbz). **(C)** Schematic diagram of the viral genomes of the recombinant reporter CTFVs. A recombinant CTFV harboring NLuc (rFlorio/VP12-2A-NLuc) or mStayGold (rFlorio/VP12-mSG) in VP12 were obtained by inserting the 2A peptide-linked NLuc or mStayGold genes at the C-terminus of VP12, respectively. These reporter CTFVs were generated using the improved reverse genetics system. **(D)** Growth kinetics of CTFV strains. Vero cells were infected with CTFV strains at a multiplicity of infection (MOI) of 0.01. The samples were collected at each time point, and viral titers of frozen–thawed samples were measured using a plaque assay. The viral growth of the reporter CTFVs (rFlorio/VP12-2A-NLuc, rFlorio/VP12-mSG) was compared with that of a recombinant wild-type CTFV (rFlorio). **(E)** NLuc activity of the cells infected with the recombinant CTFV. Vero cells were infected with CTFV strains at an MOI of 0.01. The samples were collected at each time point, and the NLuc activity of frozen–thawed samples was measured. **(F)** The localization of the mStayGold-fused VP12 in recombinant CTFV-infected Vero cells. The scale bar represents 10 μm. Significant differences were determined by performing a two-way or one-way ANOVA. The experiments were repeated at least twice to confirm the result. The experiments were repeated at least twice to confirm the result. Three biological and two technical replicates were conducted for each independent repeat. **0.005<*p*<0.01, ***p<0.005.

Finally, we attempted to rescue CTFVs expressing reporter genes inserted at the C-terminus of VP12. However, the current system, as shown in Fig 2, was unable to rescue the reporter viruses. To address this limitation, we established Expi293F cells expressing T7 RNA polymerase (Expi293F-T7), which exhibit high sensitivity to CTFV infection (Fig 1C). To compare the T7 RNA polymerase activity between Expi293F-T7 cells and BHK-T7 cells, the cells were transfected with a plasmid encoding the secretory NLuc gene driven by the T7 promoter. Remarkably, Expi293F-T7 cells demonstrated significantly higher activity compared to BHK-T7 cells (Fig 5A). Using Expi293F-T7 cells, we transfected 12 rescue plasmids along with a plasmid encoding the capping enzyme, successfully generating recombinant viruses without the need for co-culture with HeLa or Vero cells (Fig 5B). This improved system enabled the generation of reporter CTFVs by inserting NLuc and mStayGold [43] genes at the C-terminus of VP12, resulting in the rFlorio/VP12-2A-NLuc and rFlorio/VP12-mSG, respectively (Fig 5C). rFlorio/VP12-2A-NLuc expresses VP12 and NLuc separately through the 2A sequence. In contrast, rFlorio/VP12-mSG expressed a VP12 and mStayGold fusion protein to investigate the subcellular localization of VP12. While the viral growth of these reporter CTFVs was slightly reduced compared to the recombinant wild-type CTFV (Fig 5D), NLuc activity was clearly detectable upon infection with rFlorio/VP12-2A-NLuc (Fig 5E). Additionally, mStayGold-fused VP12 was observed in the cytoplasm of Vero cells infected with rFlorio/VP12-mSG (Fig 5F). The successful generation of these reporter CTFVs marks a significant advancement in facilitating vector development studies and enabling high-throughput screening applications.

## Discussion

CTFV causes a traditionally endemic disease in the western United States and Canada. A total of 59 cases of CTFV infection were reported in the United States from 2010 through 2019 [20]. However, the characteristics of CTFV are largely unknown, especially because a reverse genetics system has not been established. To establish this, we first investigated the viral growth of different CTFV isolates in cultured cells to understand basic CTFV characteristics. Most strains isolated from humans (Florio, 69V28, 83F-16B, and R97858b) grow well in Vero cells [38]. In contrast, strains (SS-18 and S5-2-50) isolated from rodents and ticks [40] tend to be less proliferative in Vero cells. These results suggest that CTFV strains isolated from humans might efficiently propagate in cells of primate origin and that the properties among strains differ depending on the host species from which they were isolated. To test this hypothesis, more isolates from different hosts should be isolated for comparative analyses.

CTFV can replicate in cell lines derived from various species, including humans, monkeys, rodents, and birds. This suggests that CTFV can infect a wide variety of species, even though it has only been reported to infect rodents, ticks, humans [3–5,7,38,40], and possibly elk, marmots, and deer [20]. In particular, avian hosts should be surveyed epidemiologically to monitor CTFV spread outside North America. Results further suggest that CTFV utilizes molecules commonly expressed across various species for infection. Of note, CTFV efficiently infected Caco-2, HeLa, and DF-1 cells within 1 h of adsorption. These findings suggest that the molecules expressed specifically and/or in large amounts in Caco-2, HeLa, and DF-1 cells play important roles in viral attachment during CTFV infection. We applied these findings to improve the CTFV reverse genetics system and found that HeLa cells strongly captured progeny viruses without inhibiting BHK-T7 cell growth, thereby increasing the rescue efficiency of recombinant CTFV strains. In MRV, which, like CTFV, is a member of the *Reoviridae* family, studies have identified receptors involved in viral infection. Junctional adhesion molecule-A was identified as a serotype-independent receptor of MRV [44]. Moreover, several receptors for MRV have been identified, and these bind to different viral proteins and demonstrate distinct viral tropisms [45–47]. However, the functions of most CTFV proteins remain largely unknown. A cell attachment protein similar to MRV Sigma1 has not yet been identified in CTFV. Moreover, no receptors for CTFV have been discovered. Therefore, further research is important to identify the receptors involved in CTFV infection, to understand viral tropism and the host range.

The CTFV reverse genetics system using BHK-T7 cells was not successful in rescuing recombinant viruses based on the overexpression of viral VP1, VP2, VP3, and VP4 proteins and RNA capping enzymes, a method previously used successfully in the TarTV reverse genetics system [12]. These results suggest that the functions of CTFV and TarTV VPs might not be fully conserved. In addition to observations in the TarTV reverse genetics system [12], the overexpression of several viral proteins in the rotavirus reverse genetics system improves rescue efficiency. In the rotavirus reverse genetic system, NSP2 and NSP5, which constitute viral inclusion bodies, enhance the rescue efficiency of recombinant rotaviruses [48,49]. Finally, recombinant viruses were successfully generated after overexpressing all viral proteins encoded by CTFV. At present, efficient viral rescue has not been achieved through the overexpression of several CTFV proteins, alone or in combination. In this study, we observed that the amino acid at position 526 of VP6 might have an important role in strain-specific differences in viral replication between the Florio and SS-18 strains. The difference in VP6 between the Florio and SS-18 strains was one amino acid (Florio: P526, SS-18: L526), but viral growth of the monoreassortant was significantly decreased. The putative functions of VP6, encoded by segment 6, include nucleotide binding and NTPase activity [7]. In the future, the effect of this amino acid mutation on the putative NTPase activity of VP6 should be investigated using virological and biochemical methods. In contrast, VP6 of strain R111081 had several amino acid variations compared to that of the Florio strain. Importantly, the 526^th^ amino acid of VP6 in strain R111081 was leucine, as in strain SS-18, although the replication ability of strain R111081 was comparable to that of the Florio strain. Therefore, conformational changes caused by multiple amino acid mutations could affect VP6 functions. This possibility should be verified through structural analysis in the future.

We successfully generated a recombinant CTFV harboring peptide-tagged VP12. We also succeeded in inserting a peptide tag into the C-terminal region of the VP12 gene of TarTV in previous studies [12]. Thus, the C-terminal region of VP12 could be a suitable candidate for the insertion of foreign genes into *Coltivirus*. However, in the genus *Cortivirus*, which possesses a 12-segment genome including TarTV, recombinant viruses expressing large-sized reporter genes have not been successfully generated. Similarly, the reverse genetics system for CTFV using BHK-T7 cells has failed to produce recombinant viruses expressing reporter genes. To address this limitation, we developed an improved system by establishing Expi293F-T7 cells, which exhibit higher T7 RNA polymerase activity compared to BHK-T7 cells. The reverse genetics system using Expi293F-T7 cells reduced the number of plasmids required for transfection to produce recombinant viruses and eliminated the need for a co-culture step. Importantly, in this study, this system enabled the rescue of two types of CTFVs expressing reporter genes. Such reporter gene-expressing CTFVs hold great potential for applications in drug screening, viral replication analysis, high-throughput specific antibody testing, and studies of viral dynamics both *in vitro* and *in vivo*, representing a significant advancement in CTFV research. We attempted to observe the subcellular localization of VP12 using recombinant viruses carrying the VP12 gene fused with a FLAG sequence. However, due to the low expression level of VP12 in infected cells, distinct localization patterns could not be analyzed. Using a reporter virus expressing the VP12 gene fused with mStayGold, VP12 was observed as dot-like structures in the cytoplasm of infected cells. Further studies are required to elucidate the detailed functions of each CTFV protein, including VP12. In addition, to further enhance the rescue efficiency of the CTFV reverse genetics system, it is essential to develop improved systems that overexpress viral proteins involved in promoting replication and minimize the number of rescue plasmids required for transfection [48–50].

In conclusion, we successfully established reverse genetics systems for CTFV. These systems also permit the introduction of the reassortment of segments, artificial peptide tags, and reporter proteins into the segmented dsRNA genomes of CTFV. The CTFV reverse genetics systems will greatly contribute to the understanding of CTFV biology, including viral replication mechanisms, pathogenesis, and transmission, as well as the development of treatments and vaccines.

## Materials and methods

### Cells

DLD-1 cells were obtained from the Cell Resource Center for Biomedical Research at the Institute of Development, Aging, and Cancer, Tohoku University, Japan. The MA104 cells were provided by Dr. Hiroshi Ushijima (Nihon University, Japan). The QT-6 cells were provided by Dr. Kazuyoshi Ikuta (Osaka University, Japan). DF-1 cells were provided by Dr. Tsuyoshi Yamaguchi (Tottori University, Japan). BHK-T7 [37], L929 (American Type Culture Collection [ATCC], Manassas, VA, USA), NIH3T3 (ATCC), MA104, CV-1 (ATCC), Caco-2 (ATCC), 293T (ATCC), MRC-5 (ATCC), DLD-1, A549 (ATCC), DF-1, QT-6, Vero (ATCC), BHK (ATCC) and Expi293F cells (Thermo Fisher Scientific, MA, USA) were maintained in Dulbecco’s modified eagle medium (DMEM; Nacalai Tesque, Kyoto, Japan) containing 5% fetal bovine serum (FBS; Thermo Fisher Scientific). HeLa cells (ATCC) were maintained in DMEM containing 10% FBS (Thermo Fisher Scientific). The cells were incubated in a 5% CO_2_ incubator at 37°C.

### Viruses

The CTFV Florio strain was obtained from the Manager of the World Reference Center for Emerging Viruses and Arboviruses, Department of Microbiology and Immunology, University of Texas Medical Branch. The other CTFV strains were generously provided by Dr. Brandy Russell of the Centers for Disease Control and Prevention. The CTFV and TarTV Kochi strains were propagated in Vero cells.

### Plaque assay

Viral titers were measured by performing plaque and focus assays. For the plaque assay, Vero cells were seeded in 24-well plates at 2 × 10^5^ cells/well. The CTFV solution was diluted in DMEM containing 2% FBS. Then, 100 μl of the diluted CTFV was inoculated in duplicate onto Vero cells and incubated at 37 °C for 1 h. After incubation, the cells were washed once and subsequently incubated with DMEM containing 2.5% FBS and 0.8% methylcellulose (FUJIFILM Wako Pure Chemical, Osaka, Japan). The plate was incubated at 37 °C for 5–7 days and the cells were subsequently fixed in PBS containing 10% formaldehyde (FUJIFILM Wako Pure Chemical) and stained with crystal violet (FUJIFILM Wako Pure Chemical). The plaques were counted manually. The viral titer was defined as PFU/ml. The standard error was calculated using GraphPad Prism 9 software (GraphPad Software, CA, USA). The high-resolution picture was taken using a Cytation 5 Cell Imaging Multimode Reader (Agilent, CA, USA) and stitched with each picture is shown in Fig 1B.

### Virus growth

Each cell line was seeded into a 48-well plate at 1 × 10^5^ cells/well. CTFV was inoculated onto the cells at a multiplicity of infection (MOI) of 0.01 for 1 h. After CTFV absorption, the cells were washed twice and incubated with 250 μl of DMEM containing 5% FBS. The time point when fresh media was added was defined as 0 hour post-infection. The cells and supernatants were frozen at different time points, and the viral titer was measured in the freeze–thawed mixture using plaque or focus assays. The supernatants were also used for the NLuc assay.

### RNA extraction

Vero cells were infected with CTFV at an MOI of 0.01 for 3 days. After incubation, the supernatant was collected and centrifuged at 1,880 × *g* for 5 min. The supernatant was then collected and centrifuged again. Next, the supernatant was ultracentrifuged at 153,700 × *g* for 1.5 h. Viral RNA was extracted from the pellet using Sepasol-RNA I Super G (Nacalai Tesque), according to the manufacturer’s instructions.

### Viral genome sequencing

Nucleotide sequences were determined via Sanger sequencing. Reverse transcription was conducted using the extracted RNA, ReverTra Ace (TOYOBO, Osaka, Japan), and Random Hexamer Primers (Thermo Fisher Scientific). PCR was performed using cDNA, KOD One (TOYOBO), and gene-specific primers. To determine the 5′ and 3′ untranslated region (UTR) sequences, the end of 3′ UTR was ligated with a self-priming anchor primer using T4 RNA ligase (Thermo Fisher Scientific). The ligated RNA was reverse transcribed using anchor primer-specific primers, and cDNA was subsequently amplified using anchor primer- and gene-specific primers [51]. The viral genome sequences of the 12 segments of CTFV Florio and segment 6 of the R111081 and SS-18 strains have been deposited into the DNA Data Bank of Japan under the accession numbers LC820581–92, LC820593, and LC820594, respectively.

### Plasmid construction

To generate pT7-CTFV-S2, 4, 5, 6, 7, 8, 9, 10, 11, and 12, the rotavirus VP1 segment in pT7-VP1SA11 [37] was replaced with cDNA of the CTFV genomes. To generate pT7-CTFV-S1 and S3, the TarTV S1 gene in pT7-TarTV-Kochi S1 [12] was replaced with CTFV S1 or S3. These ligations were conducted using NEBuilder HiFi DNA Assembly (New England Biolabs, MA, USA). The overexpression plasmid encoding each CTFV protein, downstream of the CMV promoter with codon optimization for mammals, was generated by GenScript Nucleotide Synthesis Services (GenScript, NJ, USA). The codon-optimized vaccinia virus capping genes, D1R and D12L, were generated by Eurofins Scientific Nucleotide Synthesis Services (Eurofins Scientific, Luxembourg, Luxembourg). The coding region was cloned into a pCAG plasmid, and all plasmids were transformed into competent *Escherichia coli* DH5α for amplification and collection. Since pT7-CTFV-S3 transformation resulted in critical toxicity to the DH5α strain, NEB Turbo Competent *E. coli* (High Efficiency) (New England Biolabs) was used to collect pT7-CTFV-S3 with optimized protocols. Briefly, the toxic plasmid was transformed into the NEB Turbo strain using a standard protocol. The initial culture on an LB agar plate was incubated at 30 °C for 48 h. After incubation, both large and small colonies were observed. The large colonies comprised *E. coli* harboring a frequently mutated plasmid, whereas the small colonies comprised *E. coli* transformed with pT7-CTFV-S3. To measure the T7 RNA polymerase activity, pT7-sNLuc was constructed by replacing the rotavirus genome in pT7-VP1SA11 [37] and the secreted NLuc in pNL1.3 vector (Promega, WI, USA). The NLuc gene was obtained from the pNL1.1 vector (Promega), and the mStayGold gene [43] with codon optimization for mammals was generated by GenScript Nucleotide Synthesis Services (GenScript). Porcine teschovirus 2A peptide-linked NLuc or native mStayGold genes were inserted at the C-terminal region of VP12 open reading frame in pT7-CTFV-S12, which were designated as pT7-CTFV-VP12-2A-NLuc and pT7-CTFV-VP12-mSG, respectively.

### Reverse genetics system for CTFV

BHK-T7 cells were seeded into 6-well plates at 1 × 10^5^ cells/well. The cells were transfected with 0.25 μg each of the 12 plasmids encoding CTFV genome cDNA, the 12 plasmids encoding CTFV proteins, and two plasmids encoding the vaccinia virus capping enzyme using 13 μl of TransIT-LT1 Transfection Reagent (Mirus Bio, WI, USA). The media were changed, and cells were co-cultured with 2–5 × 10^5^ Vero or HeLa cells at 6 hours post-transfection. The cells were maintained for 21–28 or 10–14 days, respectively, with appropriate medium changes (every 2–3 days when the media appeared yellow) with DMEM containing 2% FBS. The cells were frozen, thawed, and inoculated onto fresh Vero or HeLa cells. Recombinant CTFV was propagated from Vero cells using cytopathic effect.

Expi293F-T7 cells were established by infection with the lentivirus harboring mammalian codon-optimized T7 RNA polymerase gene with puromycin selection (1 μg/ml). Expi293F-T7 cells were seeded into 6-well plates at 5 × 10^5^ cells/well. The cells were transfected with 0.5 μg of each of the 12 plasmids encoding CTFV genome cDNA and two plasmids encoding the vaccinia virus capping enzyme using 14 μl of TransIT-LT1 Transfection Reagent (Mirus Bio). The cells were maintained for 10 days with appropriate medium changes (every 2–3 days when the media appeared yellow) with DMEM containing 2% FBS. The cells were frozen, thawed, and inoculated onto fresh Vero cells. Recombinant CTFV was propagated from Vero cells using cytopathic effect.

### Electrophoretic phenotyping

The dsRNA extracted from the ultracentrifuged pellets was suspended in Gel Loading Dye, Purple (6X) (New England Biolabs). The dsRNA mixture was loaded into the wells of a (Tris-borate-EDTA buffer-based) 6% polyacrylamide gel without SDS and electrophoresed. Gels were stained with GelRed Nucleic Acid Gel Stain (Biotium, CA, USA) and visualized.

### Western blotting

Vero cells were seeded into 12-well plates at 2 × 10^5^ cells/well. The recombinant CTFV strains were inoculated onto the cells at an MOI of 0.1 and incubated at 37 °C for 3 days. The supernatant was then removed, and the cells were lysed using 1 × SDS sample buffer. The lysed cells were boiled at 95 °C for 3 min and sonicated using a Bioruptor II (BM Equipment, Tokyo, Japan). The samples were then loaded onto a 5–20% e-PAGEL (Atto, Tokyo, Japan) and electrophoresed. The proteins were transferred to a polyvinylidene difluoride membrane (Millipore, Darmstadt, Germany), which was incubated with PBS containing 0.05% tween-20 (PBS-T; Nacalai Tesque) and 5% skim-milk at room temperature (20–25 °C) for 30 min. After blocking, the membrane was incubated with an anti-FLAG M2 antibody (Sigma-Aldrich, MO, USA) diluted in PBS-T containing 1% skim milk at room temperature for 1 h. The membrane was washed twice with PBS-T and subsequently incubated with horseradish peroxidase-conjugated anti-mouse IgG antibodies (Sigma-Aldrich) at room temperature for 1 h. The proteins were reacted with Chemi-Lumi One Ultra (Nacalai Tesque), and chemiluminescence was visualized using an Amersham ImageQuant 800 (GE Healthcare, IL, USA).

### Indirect fluorescent assay

Vero cells were seeded into 96-well plates at 5 × 10^5^ cells/well. The CTFV solution was diluted in DMEM containing 2% FBS, and 100 μl of the diluted CTFV was inoculated triplicately onto Vero cells and incubated at 37 °C for 15 h. The cells were then fixed and permeabilized with PBS containing 4% paraformaldehyde and 0.1% TritonX-100 (Nacalai Tesque) for 15 min. The cells were then incubated with PBS containing 0.5% skim milk (Nacalai Tesque) and the diluted primary antibody (anti-VP5, 1:2000) at 37 °C for 60 min. Subsequently, the cells were incubated with PBS containing 0.5% skim milk and the diluted secondary antibody (CF 488A goat anti-rabbit IgG, 1:2,000; Biotium) at 37 °C for 60 min. Fluorescent cells were counted manually. The CTFV-specific antibody was generated by Eurofins Scientific Peptide Synthesis and Immunization Services (Eurofins Scientific) targeting the 256–294th amino acids of CTFV VP5. The viral titer was defined as focus forming unit (FFU)/ml. The standard error was calculated using GraphPad Prism 9 software (GraphPad Software). For confocal microscopic analysis, the cells were seeded onto glass coverslips (AS ONE, Osaka, Japan) and infected. The cells were fixed and visualized using ECLIPSE Ti2 (Nikon, Tokyo, Japan).

### NLuc assay

The NLuc activity of the supernatants of frozen-thawed infected cells was measured using the Nano-Glo luciferase assay system (Promega) according to the manufacturer’s instructions.

### Statistical analyses

Two-way analysis of variance (ANOVA) was used to determine significant differences in the growth kinetics. Statistical analyses were conducted using GraphPad Prism 9 software (GraphPad Software).

## Supporting information

S1 Table Details of CTFV strains. (DOCX)
