# Supplementary material for: Establishment of reverse genetics systems for Colorado tick fever virus
Source: PLoS Pathog. 2025 Feb 14;21(2):e1012921. doi: 10.1371/journal.ppat.1012921 (PMC11828403; doi:10.1371/journal.ppat.1012921)
Supplement: S1 Table — (DOCX) [file ppat.1012921.s001.docx]

**S1 Table. Details of CTFV strains.**

| Strain name | Host | Date (YYYY/MM/DD) |
| --- | --- | --- |
| Florio | Human | 1943/1/1 |
| 71V11 | Human | 1971/1/1 |
| 69V28 | Human | 1969/1/1 |
| 83F-16B | Human | 1983/6/23 |
| R97858b | Human | 2012/7/24 |
| R111081 | Human | 2016/3/23 |
| SS-18 | Rodent | 1956/1/1 |
| S5-2-50 | Pacific coast tick | Unknown |
